# Unilateral rete mirabile in multiple intracranial arteries with ipsilateral agenesis of the internal carotid artery: a case report

**DOI:** 10.1186/s13256-023-04013-w

**Published:** 2023-06-29

**Authors:** Naoki Nitta, Atsushi Tsuji

**Affiliations:** Department of Neurosurgery, Shiga University of Medical Science, Seta-Tsukinowa-cho, Otsu, Shiga 520-2192 Japan

**Keywords:** Multiple retia mirabilia, Intraventricular hemorrhage, Internal carotid artery agenesis, Peripheral cerebral aneurysm

## Abstract

**Background:**

Rete mirabile of the cerebral artery is a rare anomaly, with most previous cases occurring in the middle cerebral artery or internal carotid artery. Here, we present the first report of unilateral rete mirabile in multiple intracranial arteries with ipsilateral internal carotid artery agenesis.

**Case presentation:**

A 64-year-old Japanese woman was brought to the emergency department of our hospital in a deep coma. Computed tomography of the head showed severe intraventricular hemorrhage with subarachnoid hemorrhage. Computed tomography angiography showed not only congenital left internal carotid artery agenesis, but also rete mirabile of the left posterior communicating artery, the left posterior cerebral artery, and the left anterior cerebral artery. This unilateral vessel anomaly complex may have contributed to the formation of a peripheral aneurysm arising from a perforating branch of the pericallosal artery, which ruptured. The patient underwent urgent bilateral external ventricular drainage, but deteriorated and was declared brain dead.

**Conclusions:**

We report the first case of unilateral rete mirabile in multiple intracranial arteries. Because the cerebral arteries in patients with rete mirabile may be vulnerable, close attention should be paid to the development of cerebral aneurysms.

## Background

Rete mirabile of the cerebral artery is a rare anomaly, and is considered to represent the persistence of the fetal arterial network of rete that normally coalesces into the distinct cerebral arteries [1]. Rete mirabile has mostly been observed in the middle cerebral artery (MCA) in the cranium and in the internal carotid artery (ICA) before it enters the cranium [1]. The frequency of rete mirabile in the MCA has been reported to be 0.11% [1]. Rete mirabile in other locations along the intracranial cerebral artery is very rare, with two cases reported in the anterior cerebral artery (ACA) and one case reported in the posterior cerebral artery (PCA) and the posterior inferior cerebellar artery [2–5].

Here, we present the first case report of unilateral rete mirabile in multiple intracranial arteries other than MCA ,concurrent with ipsilateral agenesis of the ICA. This ipsilateral vessel anomaly complex may have contributed to the formation of a peripheral aneurysm, which ruptured.

## Case presentation

**Fig. 1:**
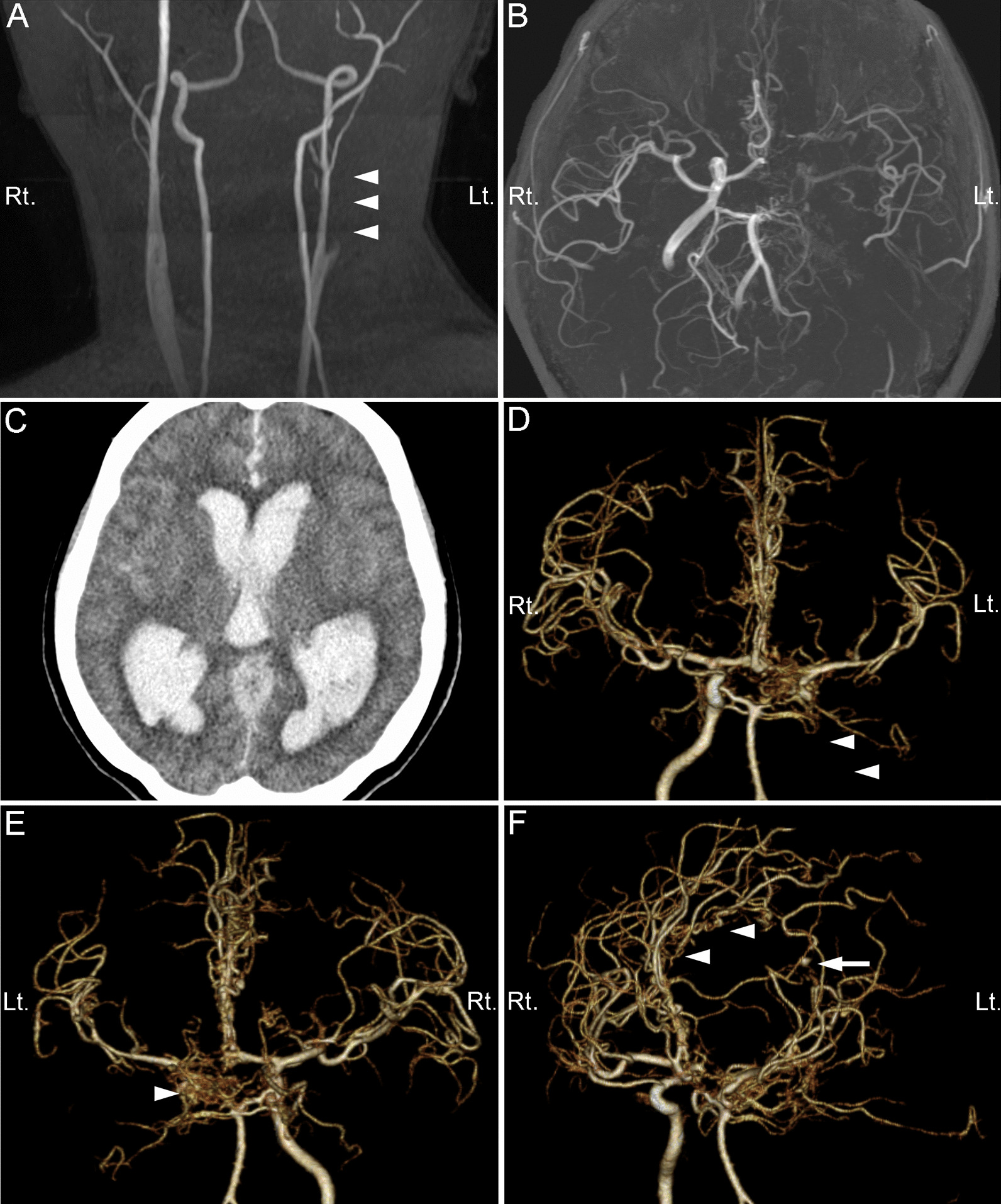
Magnetic resonance imaging of the **A** neck and **B** head 6 years before the intracranial hemorrhage described in this report. The diagnosis at that time was obliteration of the left internal carotid artery (ICA) (**A**; arrowheads) with unilateral moyamoya disease (**B**). **C** Preoperative plain computed tomography (CT) of the brain parenchyma window. **D–F** Three-dimensional CT angiography (3D–CTA) images showing anteroposterior (**D**), posteroanterior **E**, and left anterior oblique 45° **F** views. The three-dimensional CT angiography images show obliteration of the left ICA (**D**; arrowheads), rete mirabile of the left posterior communicating artery and left posterior cerebral artery (**E**; arrowhead), rete mirabile of the left anterior cerebral artery (ACA; **F**; arrowheads), and a distal ACA aneurysm (**F**; arrow). *Lt.* left; *Rt.* right

A 64-year-old Japanese woman was brought to the emergency department of our hospital in a deep coma. Six years before this admission, the patient presented at another hospital with transient right hemispatial neglect and, based on magnetic resonance imaging (MRI) and magnetic resonance angiography (MRA), was diagnosed with left ICA obliteration immediately distal to the carotid bifurcation and unilateral moyamoya disease of the ipsilateral intracranial arteries, without any aneurysm formation or cerebral infarction (Fig. 1A, B). Quantitative ^15^O gas-inhalation positron emission tomography at the time showed no apparent misery perfusion, so the patient was initially treated medically with cilostazol for probable transient ischemic attack (TIA). However, cilostazol was subsequently stopped due to increasing microbleeds in the brain on follow-up MRI. No aneurysm was detected up until the last follow-up MRA evaluation 3 months before the event described in this report.

**Fig. 2:**
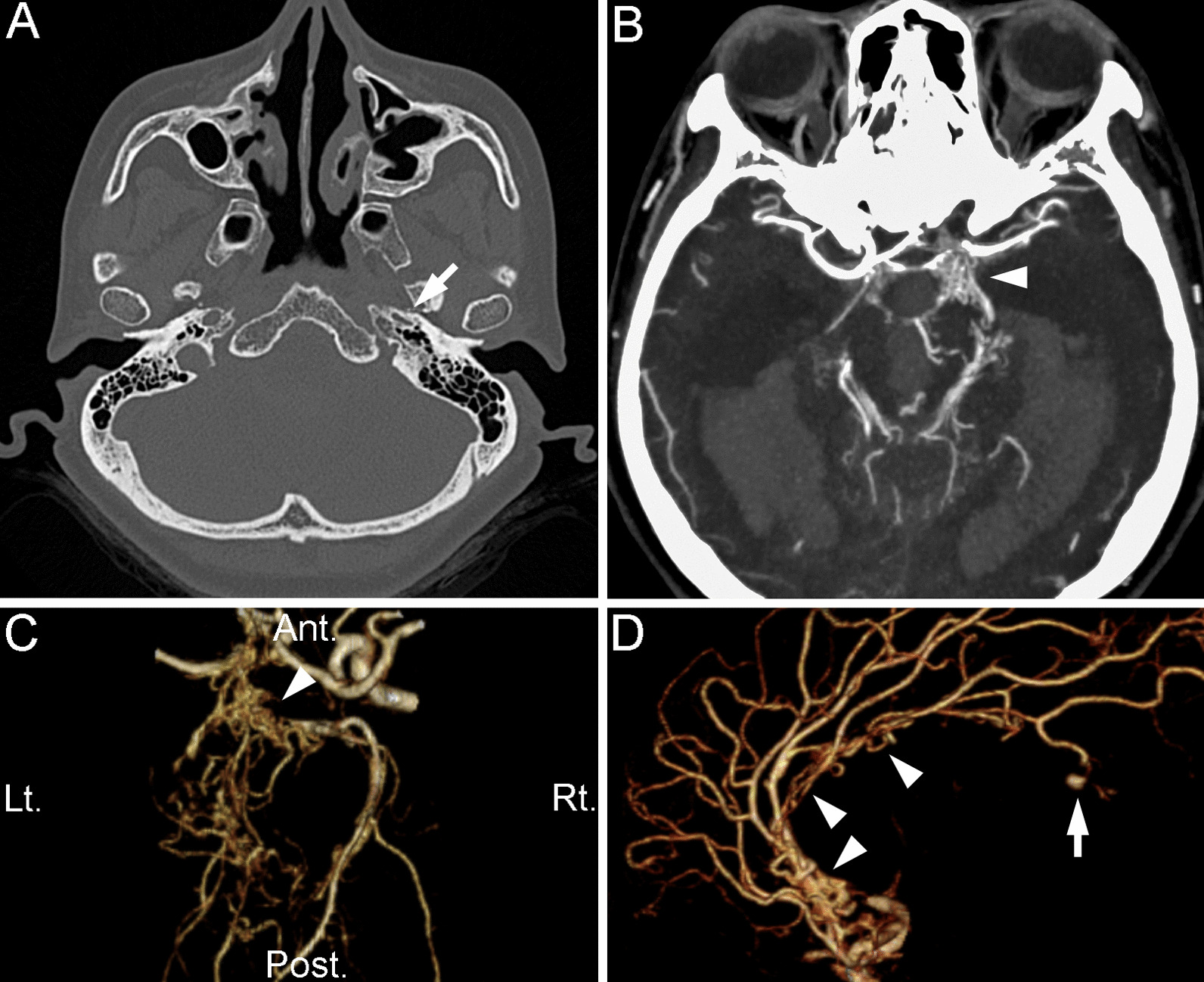
Bone window computed tomography (CT) and CT angiography (CTA). **A** Axial plain CT of the bone window. The arrow indicates the hypoplastic left carotid canal. **B** Axial CTA. The arrowhead indicates the rete mirabile of the left posterior communicating artery (PComA). **C**, **D** Three-dimensional CTA of the right–left lateral 60° view **C** and left–right lateral 15° view (**D**). **C** The arrowhead indicates the rete mirabile of the left PComA and the proximal segment of the posterior cerebral artery. **D** Arrowheads indicate the rete mirabile of the anterior cerebral artery (ACA) and the arrow indicates a distal ACA aneurysm. *Ant.* Anterior, *Lt.* left, *Post.* Posterior, *Rt.* right

Computed tomography (CT) of the head showed severe intraventricular hemorrhage with subarachnoid hemorrhage (Fig. 1C). CT angiography (CTA) showed obliteration of the left ICA with a hypoplastic carotid canal and foramen lacerum, suggesting congenital ICA agenesis rather than acquired ICA obliteration (Figs. 1D–F, 2A). The left posterior communicating artery (PComA) was not a single vessel, instead forming a vascular plexus (Figs. 1E, 2B, C). The peduncular, ambient, and quadrigeminal segments of the left PCA (Figs. 1E, 2C) and the horizontal, infracallosal, and precallosal segments of the left ACA (Figs. 1E, F 2D) looked like a network of small tortuous vessels that replaced the main trunks of the left PCA and ACA, respectively. In the distal portion of the left PCA and ACA, these aberrant networks seemed to coalesce and formed hypoplastic but definite cortical branches of the PCA and supracallosal segment of the ACA. These anomalous arteries were located in the subarachnoid space, unlike moyamoya disease or arteriovenous malformation. From these findings, we diagnosed the anomaly as rete mirabile in the PComA, PCA, and ACA. Intriguingly, the appearance of the MCA was not rete-like in this patient (Fig. 1D, E). There was a saccular aneurysm (4.3 mm in size) arising from the inferior aspect of a perforating branch of the pericallosal artery and protruding into the left lateral ventricle that was presumed to be the source of the hemorrhage (Figs. 1F, 2D). Because of her critical condition (dilatation of pupils and hypothermia), the patient immediately underwent urgent bilateral external ventricular drainage to reduce the intracranial pressure, but soon deteriorated to brain death. Thus, we did not proceed with catheter angiography and further treatment of the ruptured aneurysm. The family refused an autopsy.

## Discussion and conclusions

In the present case, rete mirabile of the PComA, the PCA, and the ACA was observed. Although rete mirabile of the cerebral arteries has been reported previously, most of these cases were rete MCA [1]. Thus, rete mirabile of the PComA, the PCA and the ACA is very rare: rete PComA has not been reported before, rete PCA has only been reported once, and rete ACA has been reported twice [2–4]. Furthermore, among all previous reports, rete mirabile has been observed in only one cerebral artery. This paper is the first to report rete mirabile of several cerebral arteries. In the present case, ipsilateral ICA agenesis was accompanied by a hypoplastic left carotid canal, suggesting congenital ICA agenesis rather than acquired ICA obliteration. The vascular anomaly in our patient may have resulted from the maldevelopment of a broad extent of unilateral hemispheric cerebral arteries.

A rete mirabile, which is also referred to as a twig-like cerebral artery, may be defined as a vascular network that interrupts the continuity of a vessel. Although rete mirabile is a rare anomaly of the cerebral artery, comorbid aneurysm formation and rupture have been reported in some patients. In a review of retia mirabilia with associated aneurysms [6], there were 13 cases of rete MCA and two cases of rete posterior inferior cerebral artery associated with aneurysms. Thus, most retia mirabilia associated with aneurysms have been observed in the MCA. Only in one case of rete mirabile of the ACA was a ruptured aneurysm observed, and this existed outside the collateral network [3, 4]. No aneurysm was observed in the only previous case of rete mirabile of the PCA [2]. In the present case, the aneurysm arose from a perforating branch of the pericallosal artery, where rete mirabile was not observed. In a review of 15 cases of nonmoyamoya collateral networks with associated aneurysms [6], there were five cases of an aneurysm outside the collateral network, suggesting that vulnerability or abnormal hemodynamic stress occurs not only at the site of the rete mirabile itself [7], but also in distinct cerebral arteries that connect to the rete mirabile. Although MRA follow-up did not detect the formation of a peripheral aneurysm in the present case, attention should be paid to the development of cerebral aneurysms not only at the site of rete mirabile, but also beyond it. If, in the present case, the patient had undergone CTA or catheter angiography at the time of initial TIA, an early diagnosis of multiple retia mirabilia and aneurysm may have been made. However, if a patient is asymptomatic, treatment interventions may not be performed at first because the aneurysm might be small and because it may not be possible to infer the likelihood of its rupture and to access it via endovascular surgery. A practical strategy after detecting an aneurysm is to clip it after having confirmed its rapid growth by close follow-up. However, even if we could have followed this strategy in the present case, the outcome may have been the same.

Considering the development of the cranial arteries, the abnormal vascular network in our patient could have developed during the fetal stage. As suggested previously, normal fusion or regression of the primordial blood–vessel plexus of the head during fetal development would result in the disappearance of the blood–vessel plexus, whereas incomplete fusion or regression may result in a fenestration or plexiform remnant [3,[8]. The development of cranial arteries in human embryos was described in detail by Padget (Table 1) [8]. Based on that description, we speculate that the persistence of rete mirabile of the caudal division of the embryonic ICA (the future adult PComA and adult PCA) and obliteration of the entire proximal segment of the embryonic ICA is predetermined at or before the developmental periods corresponding to embryo sizes 4–12 mm, and that the persistence of the rete mirabile of the distal segment of the cranial division of the embryonic ICA (the future adult ACA) is predetermined before or at the time when embryos are 12–18 mm [8]. Because the primitive MCA emerges from the second segment of the cranial division of the embryonic ICA (Table 1) [8], only that segment developed normally in our patient, but we cannot explain why.

**Table 1:** Correlation between human embryo size and development of the cranial arteries

Embryo size (mm)	Characteristic features of embryos [7]
4	The primitive ICA has the appearance of having just emerged from a plexus and the cranial division [future terminal carotid segment (C1) of the ICA, future MCA, and future ACA] and caudal division (future PComA and PCA) of the embryonic ICA have been established
5–6	The caudal division has formed an anastomosis with the cranial end of the longitudinal neural artery (the future basilar artery) at the mesencephalon and has thereby become the definitive PComA
7–12	The future peduncular segment of the adult PCA has been established at the caudal end of the embryonic PComA (the stem of the embryonic PCA), and diencephalic and mesencephalic branches (future distal segments of the adult PCA) have emerged
12–14	The most proximal segment of the cranial division of the embryonic ICA gives off the anterior choroidal artery as its first branch; the second collateral is the MCA, and the next segment is the ACA in an early stage of formation The cerebral arteries are far less plexiform and the origin of many adult arteries is already indicated at this stage
16–18	The embryonic ACA has become a direct continuation of the cranial division of the embryonic ICA

*ACA* anterior cerebral artery, *ICA* internal carotid artery, *MCA* middle cerebral artery, *PCA* posterior cerebral artery, *PComA* posterior communicating artery

A limitation of this case report is the absence of catheter angiography, which may be superior to CTA for the visualization of unilateral rete mirabile in multiple intracranial arteries and peripheral aneurysms. In the present case, the patient’s condition was so severe that we could not proceed to catheter angiography. Although the resolution of CTA is lower than that of catheter angiography, in our patient it did produce images of sufficiently high resolution to enable a diagnosis of rete mirabile and the identification of a peripheral aneurysm. Another limitation of this report is the lack of an autopsy.

In conclusion, we have reported a case of rete mirabile of the left PComA, ACA, and PCA with ipsilateral ICA agenesis. This is the first report of unilateral rete mirabile in multiple intracranial arteries. Because the cerebral arteries in patients with rete mirabile may be vulnerable, attention should be paid to the development of cerebral aneurysms.

## Data Availability

The data that support the findings of this study are available on request from the corresponding author.

## Abbreviations

ACA: Anterior cerebral artery
CT: Computed tomography
CTA: CT angiography
ICA: Internal carotid artery
MCA: Middle cerebral artery
MRA: Magnetic resonance angiography
MRI: Magnetic resonance imaging
PCA: Posterior cerebral artery
PComA: Posterior communicating artery
TIA: Transient ischemic attack
